# The “Forgotten” Subtypes of Breast Carcinoma: A Systematic Review of Selected Histological Variants Not Included or Not Recognized as Distinct Entities in the Current World Health Organization Classification of Breast Tumors

**DOI:** 10.3390/ijms25158382

**Published:** 2024-08-01

**Authors:** Nektarios I. Koufopoulos, Ioannis Boutas, Abraham Pouliakis, Menelaos G. Samaras, Christakis Kotanidis, Adamantia Kontogeorgi, Dionysios T. Dimas, Argyro-Ioanna Ieronimaki, Danai Leventakou, Aris Spathis, Magda Zanelli, Andrea Palicelli, Maurizio Zizzo, Dimitrios Goutas, Ioannis S. Pateras, Ioannis G. Panayiotides

**Affiliations:** 1 Second Department of Pathology, Medical School, National and Kapodistrian University of Athens, Attikon University Hospital, 15772 Athens, Greece; apouliak@med.uoa.gr (A.P.); ioagpan@med.uoa.gr (I.G.P.); 2 Breast Unit, Rea Maternity Hospital, Palaio Faliro, 17564 Athens, Greece; 3 Third Department of Obstetrics and Gynecology, Medical School, National and Kapodistrian University of Athens, Attikon University Hospital, 15772 Athens, Greece; 4 Breast Unit, Athens Medical Center, Psychiko Clinic, 11525 Athens, Greece; 5 Pathology Unit, Azienda Unita Sanitaria Locale-IRCCS di Reggio Emilia, 42123 Reggio Emilia, Italy; 6 Surgical Oncology Unit, Azienda USL-IRCCS di Reggio Emilia, 42122 Reggio Emilia, Italy; maurizio.zizzo@ausl.re.it; 7 Clinical and Experimental Medicine PhD Program, University of Modena and Reggio Emilia, 41121 Modena, Italy; 8 First Department of Pathology, Medical School, National and Kapodistrian University of Athens, 11527 Athens, Greece

**Keywords:** metaplastic carcinoma, metaplastic carcinoma with melanocytic differentiation, lymphoepithelioma-like carcinoma, lymphoepithelioma-like breast carcinoma, signet-ring cell carcinoma, breast carcinoma with osteoclast-like giant cells

## Abstract

Breast carcinoma is the most common cancer in women. Nineteen different subtypes of breast carcinomas are recognized in the current WHO classification of breast tumors. Except for these subtypes, there are a number of carcinomas with special morphologic and immunohistochemical features that are not included in the 5th WHO classification, while others are considered special morphologic patterns of invasive breast carcinoma of no special type. In this manuscript, we systematically review the literature on four different subtypes of invasive breast carcinoma, namely lymphoepithelioma-like breast carcinoma, breast carcinoma with osteoclast-like giant cells, signet-ring breast carcinoma, and metaplastic breast carcinoma with melanocytic differentiation. We describe their clinicopathological characteristics, focusing on the differential diagnosis, treatment, and prognosis.

## 1. Introduction

Breast cancer is the most common cancer in women (accounting for 24.5% of all carcinomas). It is the leading cause of mortality (15.5%) from cancer among female patients, with a declining tendency, especially in patients of younger age (less than 50 years old) [1,2]. It will affect 310,720 women and 2800 men in 2024 [3]

The current World Health Organization Classification of Breast Tumors (5th edition) recognizes nineteen subtypes of invasive breast carcinoma (IBC) including IBC of no special type [4], invasive lobular carcinoma (ILC) [5,6], malignant adenomyoepithelioma [7], invasive papillary carcinoma [8], tubular carcinoma [9,10], cribriform carcinoma [11], mucinous carcinoma [12], mucinous cystadenocarcinoma [13], invasive micropapillary carcinoma [14,15], carcinoma with apocrine differentiation [16,17], metaplastic carcinoma [18], adenoid cystic carcinoma [19], acinic cell carcinoma [20], secretory carcinoma [21,22], mucoepidermoid carcinoma [23,24], polymorphous adenocarcinoma [25,26], tall cell carcinoma with reversed polarity [27], neuroendocrine tumor [28,29], and neuroendocrine carcinoma [30,31]. The most frequent subtype of IBC is of no special type, accounting for the majority (60–80%) of cases [4]. ILC is the most common special subtype, accounting for 10–15% of IBCs. It displays special morphological, immunohistochemical, clinical, and imaging features and a different metastatic pattern compared to IBCs of NST [32,33]. The other subtypes have specific architectural, cytological, immunohistochemical, and molecular characteristics and have either a better (mucinous, tubular, cribriform, and tall cell carcinoma with reversed polarity) or worse (micropapillary, and most subtypes of metaplastic carcinoma) biologic behavior [4]. So for therapeutic and prognostic reasons the correct distinction of breast carcinoma subtypes is very important.

Apart from the recognized subtypes of IBC, there are a number of IBCs that are either considered as special morphologic patterns of IBC of NST, such as breast carcinoma with osteoclast-like giant cells (BC-OGC), signet-ring cell carcinoma of the breast (SRCC-B), and metaplastic breast carcinoma (MBC) with melanocytic differentiation or not mentioned at all in the current WHO classification such as lymphoepithelioma-like breast carcinoma (LELC-B). We choose to focus on these subtypes due to their rarity and because, to our knowledge, this is the first review on any of these subtypes. This manuscript systematically reviews the literature on these four entities and describes their clinical, morphological, and immunohistochemical characteristics, focusing on the differential diagnosis, treatment, and prognosis.

## 2. Methods

In all cases, we performed a systematic review of the literature according to the PRISMA (“Preferred Reporting Items for Systematic Reviews and Meta-Analyses”) guidelines (http://www.prismastatement.org/; accessed on 29 March 2024).

### 2.1. Systematic Review of the Literature of Lymphoepithelioma-like Breast Carcinoma

**Figure 1:**
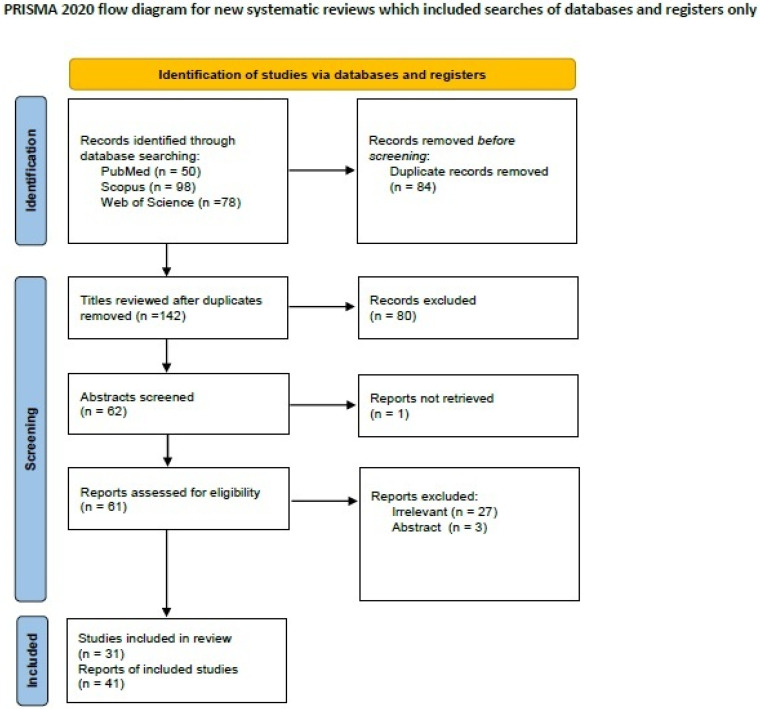
PRISMA 2020 flowchart showing the search strategy, excluded studies, and finally included reports of lymphoepithelioma-like breast carcinomas.

We searched for LELC-Bs on PubMed (all fields; 50 results; https://pubmed.ncbi.nlm.nih.gov, accessed on 29 March 2024), Scopus (Title/Abstract/Keywords; 98 results; https://www.scopus.com/home, accessed on 29 March 2024), and Web of Science (all fields; 78 results; https://login.webofknowledge.com, accessed on 29 March 2024) using the terms ((“lymphoepithelioma”) AND (“breast” OR “mammary”) AND (“carcinoma”)). We included all primary articles and case reports in the English language describing LELC-B. We excluded abstracts of medical conferences, previous review articles, and articles describing cases with unclear diagnoses and missing or too much aggregated data. Three authors [I.B., D.D. and M.G.S.] performed the literature review and collected data. Discrepancies were corrected in consensus. After applying inclusion and exclusion criteria, 31 manuscripts describing 41 cases of LELC-B remained for data extraction [34,35,36,37,38,39,40,41,42,43,44,45,46,47,48,49,50,51,52,53,54,55,56,57,58,59,60,61,62,63,64]. A PRISMA flow chart with a summary of search results is shown in Figure 1.

### 2.2. Systematic Review of the Literature on Breast Carcinoma with Osteoclast-like Giant Cells

**Figure 2:**
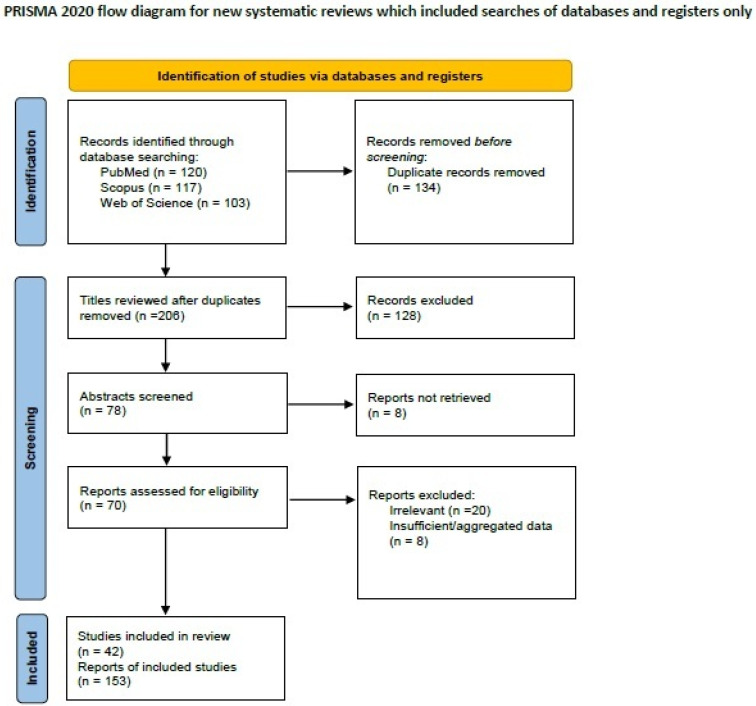
PRISMA 2020 flowchart showing the search strategy, excluded studies, and finally included reports of breast carcinomas with osteoclast like giant cells.

We searched the literature for BC-OGCs on PubMed (all fields; 120 results; https://pubmed.ncbi.nlm.nih.gov, accessed on 29 March 2024), Scopus (Title/Abstract/Keywords; 117 results; https://www.scopus.com/home, accessed on 29 March 2024), Web of Science (all fields; 103 results; https://login.webofknowledge.com) accessed on 29 March 2024) using the terms ((“breast” OR “mammary”) AND (“cancer” OR “carcinoma”) AND (“osteoclast” AND “like” AND “giant cells”)). We included all primary articles and case reports in English describing BC-OGCs. Abstracts of medical conferences and previous reviews were excluded. Papers reporting tumors of uncertain diagnosis or studies with scant or too much aggregated data were excluded. Three authors [I.B., A.K. and M.G.S.] performed the literature review and collected data. Discrepancies were corrected in consensus. After applying inclusion and exclusion criteria, 43 manuscripts describing 153 cases of BC-OGCs remained for data extraction [65,66,67,68,69,70,71,72,73,74,75,76,77,78,79,80,81,82,83,84,85,86,87,88,89,90,91,92,93,94,95,96,97,98,99,100,101,102,103,104,105,106]. A PRISMA flow chart with a summary of search results is shown in Figure 2.

### 2.3. Systematic Review of the Literature on Signet-Ring Cell Breast Carcinoma

**Figure 3:**
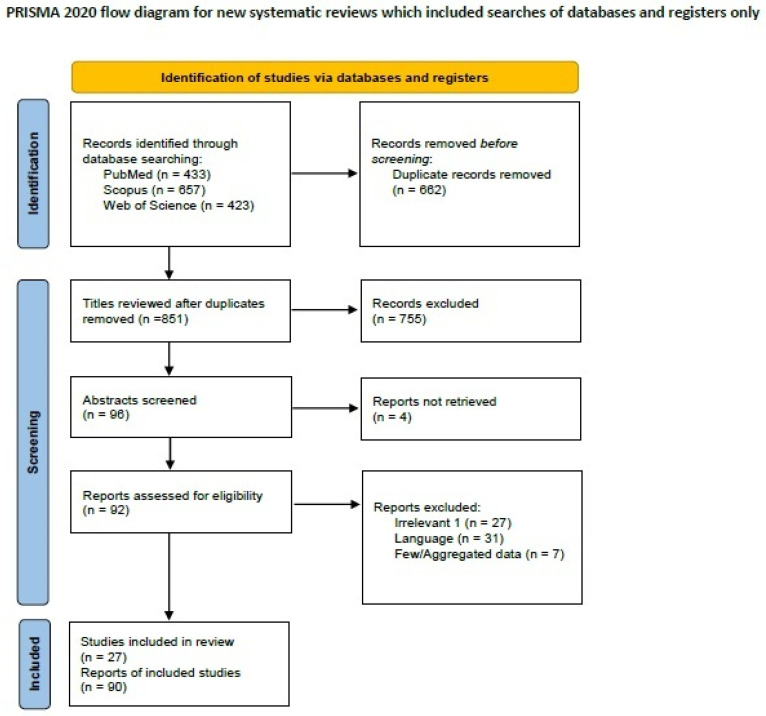
PRISMA 2020 flowchart showing the search strategy, excluded studies, and finally included reports of signet-ring cell breast carcinomas.

We searched the literature for SRCC-B on PubMed (all fields; 433 results; https://pubmed.ncbi.nlm.nih.gov, accessed on 29 March 2024), Scopus (Title/Abstract/Keywords; 657 results; https://www.scopus.com/home, accessed on 29 March 2024), Web of Science (all fields; 423 results; https://login.webofknowledge.com, accessed on 29 March 2024) research, and the terminologies ((“signet-ring”) AND (“breast” OR “mammary”) AND (“carcinoma”)). We included all primary articles and case reports in the English language describing SRCCs, such as interventional, observational, prospective, and retrospective studies and case reports. Abstracts of medical conferences, editorials, preliminary studies with animal models, and previous reviews were excluded. Papers reporting tumors of uncertain diagnosis or studies with scant or too much aggregated data were excluded. Three authors [A.K., D.G. and M.G.S.] performed the literature review and collected data. Discrepancies were corrected in consensus. After applying inclusion and exclusion criteria, 27 manuscripts describing 90 signet ring cell carcinoma cases remained for data extraction [107,108,109,110,111,112,113,114,115,116,117,118,119,120,121,122,123,124,125,126,127,128,129,130,131,132,133]. A PRISMA flow chart with a summary of search results is shown in Figure 3.

### 2.4. Systematic Review of the Literature on Metaplastic Breast Carcinoma with Melanocytic Differentiation

**Figure 4:**
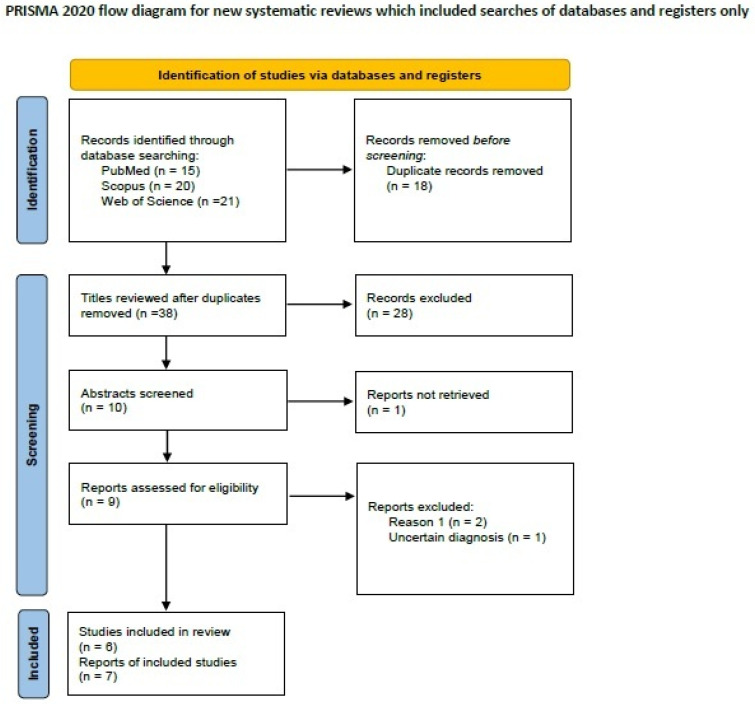
PRISMA 2020 flowchart showing the search strategy, excluded studies, and finally included reports of metaplastic carcinomas with melanocytic differentiation.

We searched the literature for MBCs with melanocytic differentiation on PubMed (all fields; 15 results; https://pubmed.ncbi.nlm.nih.gov, accessed on 29 March 2024), Scopus (Title/Abstract/Keywords; 20 results https://www.scopus.com/home, accessed on 29 March 2024), Web of Science (all fields; 21 results; https://login.webofknowledge.com, accessed on 29 March 2024), using the terms ((“breast”) AND (“carcinoma”) AND (“melanocytic differentiation”)). We included all primary articles and case reports in English describing metaplastic breast carcinoma with melanocytic differentiation, as well as original articles and case reports. Abstracts of medical conferences and manuscripts reporting cases in which the diagnosis was uncertain, or the studies with insufficient data were excluded. Two authors [D.D. and D.G.] performed the literature review and collected data. Discrepancies were discussed and corrected in consensus. After applying inclusion and exclusion criteria, six manuscripts describing seven cases of MBC with melanocytic differentiation remained for data extraction [134,135,136,137,138,139]. A PRISMA flow chart with a summary of search results is shown in Figure 4.

## 3. Results and Discussion

### 3.1. Lymphoepithelioma-like Breast Carcinoma

#### 3.1.1. Demographic and Clinicopathological Features

Lymphoepithelioma-like breast carcinoma (LELC-B) represents less than 1% of all breast carcinomas. It is the mammary analog of the nasopharyngeal lymphoepithelioma. Kumar and Kumar initially described this entity in 1994 [34]. Tumors with similar morphology have been described in several different organs, including the esophagus, stomach, colon, hepatobiliary tract, kidney, ureter, urinary bladder, prostate, larynx, trachea, lungs, major and minor salivary glands, lacrimal gland, orbital adnexa, thymus, thyroid, uterine cervix, vagina, vulva, and skin [59].

Lymphoepitheliomas of the nasopharynx, as well as lymphoepithelioma-like carcinomas (LELC) of the salivary glands, stomach, and thymus, have been associated with Epstein-Barr virus (EBV) infection. In contrast, LELCs of the skin, uterine cervix, oral cavity, and urinary bladder have not been related to EBV. EBV has not been detected in all LELC-B cases tested with various methods, including in situ hybridization [35,36,39], polymerase chain reaction [35], and immunohistochemistry [38]. Three cases have been associated with HPV infection [43,48,59].

In our review, we found 31 manuscripts describing 41 cases of LELC-B. Patient age ranged from 37 to 69 years (mean 52.9 years). The mean tumor size was 24.7 mm (range 10–45 mm). Among the cases that reported nodal status, ten showed lymph node metastasis [36,37,38,42,47,52,56,57,59,62].

The demographic, clinical, pathological and treatment features of the reported cases are displayed in Supplementary Tables S1 and S2.

#### 3.1.2. Imaging Findings

Imaging findings are not specific for this type of tumor. LELCs may present on mammography either as a well-circumscribed [35,38], irregular [43,47,48,60,62] lobulated [49,53,64], or ill-defined [52] mass. They may have indistinct [44,45] or microlobulated [54,55] margins or appear as a hypoechoic mass on ultrasound [42,44].

#### 3.1.3. Histological Findings and Differential Diagnosis

On gross examination, these tumors may have a nodular appearance [35,54,57], lobulated [42], well-circumscribed [37,39,45,51,59,64], or have infiltrating margins [38,46]. Occasionally, no discrete tumor could be identified [40].

**Figure 5:**
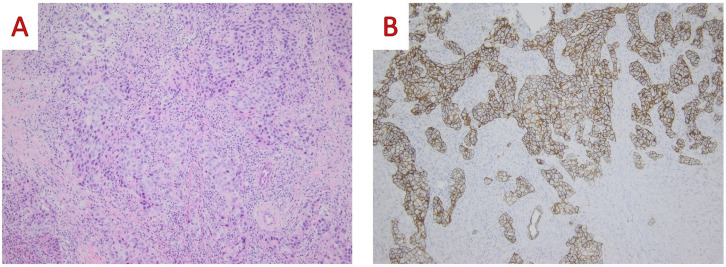
(**A**) On low power examination, tumor cells are intimately admixed with a dense lymphoid stroma (Hematoxylin and Eosin, H&E; ×100); (**B**) On immunohistochemical examination, a immunopositivity in Cytokeratin AE1/AE3 demonstrates the epithelial nature of tumor cells (Cytokeratin AE1/AE3 mouse monoclonal AE1/AE3, Dako ×100) (original, previously unpublished photos).

Microscopically, LELC-Bs are characterized by nests, cords, or syncytial appearance (Regaud pattern) or isolated tumor cells (Schminke pattern) associated with a dense lymphoid stroma stained positive for CD3 (T cell) and CD20 (B cell) markers. Tumor necrosis was identified in a few cases [36,41,50]. Some authors have reported a lobular morphology [34,35,38,40] associated in some of these cases with lobular carcinoma in situ [34,35,38], atypical lobular hyperplasia [40], and pagetoid spread [35]. None of the cases was associated with ductal carcinoma in situ. Three of the cases there were associated with sclerosing lymphocytic lobulitis [37,51,52]. Glandular differentiation on electron microscopy was reported by Kurose et al. [41]. The majority of LELC-Bs have a triple-negative phenotype [60]. LELC-Bs mimics undifferentiated nasopharyngeal carcinoma which is associated with Epstein Barr virus (EBV); so far none of the reported cases with LELC-B are associated with EBV [60]. A case of LELC-B is displayed in Figure 5.

The differential diagnosis of LELC-B includes invasive breast carcinoma with a medullary pattern (medullary carcinoma), lymphocyte-rich invasive carcinoma of ductal or lobular type, and lymphoproliferative disorders such as non-Hodgkin’s and Hodgkin’s lymphoma.

LELC-B is probably underrecognized because it shares histological features similar to lymphocyte-predominant breast carcinoma (LPBC).

Invasive breast carcinoma with medullary pattern is no longer recognized as a distinct entity by the current WHO classification but rather as a special morphologic pattern of IBC of NST. Histologically, it is defined as a carcinoma that fulfills a number of criteria proposed by Ridolfi et al. These criteria include complete microscopic circumscription, extensive syncytial growth pattern (more than 75%), moderate to marked lymphoid stromal infiltration, high nuclear grade and increased number of mitotic figures, lack of in situ component, or gland formation [140]. Lymphocyte-rich breast carcinoma (LPBC) is a carcinoma with at least 50–60% inflammatory stroma [141]. The difference between LELC-B and LPBC is the quantity of lymphocytes, which in LELC-B obscures the neoplastic cells in contrast to LPBC. Also, some architectural and cytological features may help make the correct diagnosis [36].

It could be challenging to differentiate the single-cell pattern of LELC-B from non-Hodgkin’s or Hodgkin’s lymphoma. In some previously reported cases, non-Hodgkin’s lymphoma was the principal differential diagnosis [37,39]. It is essential to know that a number of cases were initially misdiagnosed as non-Hodgkin’s lymphoma [36,49,56]. In these cases, immunohistochemical markers such as a panel of keratins (CKAE1/AE3, CK8/18 CK19 and CK7) together with selected lymphoid markers (CD45, CD3, CD20, CD15, CD30) may help. Positive staining for markers of epithelial differentiation paired with negative staining for lymphoid markers will provide the diagnostic solution in difficult cases. Also of note is the CD117 positivity, either diffuse or focal in few cases [49,51,57,63,64].

#### 3.1.4. Molecular Studies

To the best of our knowledge there have been no studies addressing the molecular profile of these tumors.

#### 3.1.5. Treatment

Surgical treatment data was reported in 39/41 (95.1%) cases. In 16/39 (41%) [34,36,38,41,45,46,55,56,60,61,63,64] cases treatment consisted of mastectomy, while 23/39 (59%) [35,36,37,39,40,42,43,44,47,48,49,51,52,54,56,57,58,59,62] patients underwent breast-conserving surgery. Details regarding adjuvant treatment were mentioned in 33/41 (80.5%) [35,36,38,39,41,44,45,46,48,49,50,52,54,55,56,57,59,61,62,63] cases. Adjuvant or neoadjuvant chemotherapy was offered in 20/33 (60.6%) [38,41,44,45,46,48,50,52,54,55,56,57,59,62,63] cases, hormonal therapy in 3/33 (9%) [39,54,61], and radiotherapy in 17/33 (51.5%) [35,36,39,41,44,48,49,52,54,56,57,59,61,62,63] cases. Two patients refused the proposed chemotherapeutic regimen [49,64]. Due to the rarity of this entity, there is no evidence of optimal treatment. Patients with LELC-B have not been included in randomized controlled trials. Therefore, their management is based on anecdotal cases or published case reports [54].

#### 3.1.6. Outcome

Follow-up information was available in 33/41 (80.5%) [34,35,36,37,38,39,41,44,45,46,48,49,50,52,54,56,57,59,60,61,62,63,64] cases Most patients were alive without evidence of disease in a timeline ranging from 3 to 103 months [34,35,36,37,38,39,44,45,48,49,50,52,56,57,59,60,61,62,63,64] (median: 24 months). Two cases developed contralateral carcinoma (LELC-B in one of them) 36 and 53 months later [36,56]. One case showed a parasternal mass after four months and metastasis to the parasternal lymph node and lung 19 months later [41]. Another case had a local recurrence after 18 months without evidence of distant metastasis [46]. None of the patients died during the follow-up time.

### 3.2. Breast Carcinoma with Osteoclast-like Giant Cells

#### 3.2.1. Demographic and Clinicopathological Features

The presence of osteoclast-like giant cells (OGC) has been well documented in several tumors including undifferentiated carcinoma of the pancreas [142], small cell lung carcinoma [143], gastric carcinoma [144], hepatocellular carcinoma [145], urothelial carcinoma [146], renal cell carcinoma [147], anaplastic thyroid carcinoma [148], neuroendocrine tumor of the jejunum [149], ductal carcinoma of the parotid gland [150], leiomyosarcoma [151], cervical squamous cell carcinoma [152], epithelioid haemangioendothelioma [153], and melanoma [154].

In mammary pathology giant cells have been reported as isolated findings in mammary stroma [155], in fibroadenomas [156], phyllodes tumors [157], DCIS [158], and invasive breast carcinomas [65].

BC-OGC represents about 0.5–1% of all breast carcinomas. In our review, we found 42 articles describing 153 BC-OGC cases. Mean age was 50.2 years (range 27–84 years). The mean tumor size was 27.9 mm (range 4–109 mm). Concerning histological subtype 100/153 (65.3%) [65,66,67,69,70,73,75,77,79,80,82,84,85,88,89,90,91,93,95,96,97,99,101,102,103,104,105,106] were invasive breast carcinomas of no special type, 22/153 (14.4%) [65,70,82,85,93,102] were mixed carcinomas, 13/153 (8.5%) [65,74,83,88,102,103], metaplastic carcinomas, 9/153 (5.9%) [68,72,81,85] invasive cribriform carcinomas, 5/153 (3.2%) [71,76,78,98,100], invasive lobular carcinomas, 2/153 (1.3%) [87,94] neuroendocrine, 1/153 (0.6%) [86] pleomorphic carcinoma and 1/153 (0.6%) [92] adenoid cystic carcinoma. The summary of the demographic, clinicopathological and treatment features of these cases is displayed in Supplementary Tables S3 and S4.

#### 3.2.2. Imaging Findings

The imaging features of BC-OGCs are not pathognomonic, showing increased peripheral vascularity at ultrasonography without any other distinguishing features [105].

#### 3.2.3. Histological Findings and Differential Diagnosis

**Figure 6:**
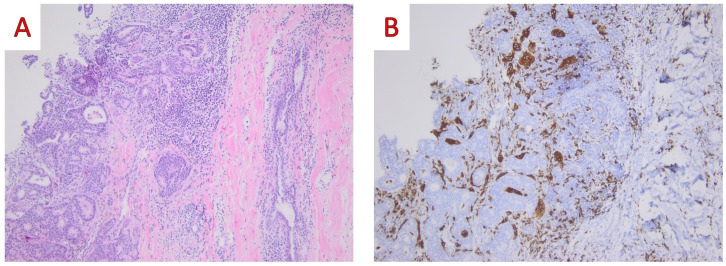
(**A**) On low power examination, malignant epithelial cells are lying close to large multinucleated giant cells with abundant cytoplasm and numerous centrally located nuclei (Hematoxylin and Eosin, H&E; ×100); (**B**) On immunohistochemical examination CD68 (PGM1) immunostaining highlights the presence of osteoclast-like giant cells (CD68 mouse monoclonal PG-M1, Dako ×100) (original, previously unpublished photos).

Grossly, BC-OGC presents as well-circumscribed round tumors with a characteristic dark brown or red-brown “rusty” color [68,105] On microscopic examination, the neoplastic cells may show several different histologic patterns, including tubular, cribriform, papillary, and solid; they may be arranged in a single file pattern or float in extracellular mucin. Occasionally, they may show neuroendocrine differentiation [87,94]. Less frequently, tumor cells may show clear cell features [96]. Importantly, the distinguishing feature of these carcinomas is the presence of large multinucleated giant cells with abundant cytoplasm and numerous centrally located nuclei expressing the histiocytic marker CD68, while there are negative for the epithelial marker CKAE1/AE3. The tumor stroma may also be infiltrated by macrophages, lymphocytes, and monocytes. Of note is the presence of extravasated red blood cells and hemosiderin deposits [104]. Tumor grade was reported in 122 cases. In one case, tumor grade was unknown; 46 cases were grade I, 54 were grade II, and 21 were grade III. Figure 6 shows the histological characteristics of BC-OGC.

The distinguishing histological characteristic of BC-OGC is the presence of distinct stromal features such as an inflammatory hypervascular stroma, rich in fibroblasts, with extravasated erythrocytes, monocytes, and lymphocytes together with histiocytes, and multinucleated giant cells [105]. The carcinoma associated with these stromal components is usually a carcinoma of no special type, but several other histotypes may be present. With this in mind, the spectrum of differential diagnoses of BCOGC may be broad. The main differential diagnosis should be made with pleomorphic carcinoma due to the presence of tumor giant cells in the latter. In contrast to osteoclast-like giant cells, which are bland-looking, pleomorphic giant cells are highly atypical, pleomorphic, and bizarre. Osteoclast-like giant cells are immunohistochemically positive for CD68 and negative for epithelial markers. The opposite is true for the giant cells of pleomorphic carcinoma.

#### 3.2.4. Molecular Studies

Cyrta et al. performed sequencing in thirteen (*n* = 13) BC-OCG cases demonstrating hotspot mutations in PIK3CA in four cases and to a lesser extent truncating mutations in MAP3K1 and MAP2K4 (one each), hotspot activation mutation in AKT1 (*n* = 1), in-frame deletion in PIK3R1 (*n* = 1), frameshift and splice site mutation in TP53 (*n* = 1), BRAF hot spot V600E mutation (*n* = 1), GNAS and HRAS hotspot mutations (*n* = 2) and one potentially pathogenic PTEN variant (*n* = 1) [102].

Interestingly, in the same study transcriptomic analysis in BC-OCG along with IBC (NST) without OCG cases, revealed a separation of two gene expression profiles. BC-OCGs demonstrated significant overexpression of genes associated with OCG differentiation including TNFSF11 (encoding RANK-L that promotes osteoclast formation), TNFSFR11A (encoding RANK, the receptor of RANK-L), CSF1 (encoding the cytokine M-CSF) and CSF1R compared with IBC (NST) without OCG [102]. On the other hand, the levels of OPG (encoding osteoprotegerin which suppresses RANK-L/RANK axis) were significantly lower in BC-OCG versus IBC (NST) without OCG. Immunohistochemical analysis verified gene expression profile, showing increased immunopositivity of cancer cells for RANK-L in BC-OCG [102]. Interestingly, OGCs did not express RANK-L.

#### 3.2.5. Treatment

In 105/153 (68.6%) [66,67,68,69,70,71,72,73,74,75,76,77,78,79,80,81,83,84,86,88,90,91,92,93,95,98,99,100,101,102,104,105,106] cases there was information regarding surgical treatment 65/105 (61.9%) [66,67,68,69,70,71,75,76,77,78,79,80,83,86,92,93,98,99,102] patients were treated with mastectomy and 38/105 [72,74,84,88,90,91,95,100,101,102,104,105,106] (36.2%) with a breast-conserving surgical procedure. In 2/105 (1.9%) cases the patients did not undergo surgery but instead a tru-cut [73] and a core biopsy [81] were performed respectively.

Detailed information concerning adjuvant therapy was provided for 45/153 (29.4%) [72,73,74,79,80,90,91,95,98,99,100,101,102,104,106] patients. Chemotherapy was administered in 19/45 (42.2%) [79,80,90,91,99,101,102,104,106] patients, two of them in the neoadjuvant setting, and hormonal therapy in 28/45 (62.2%) [73,74,90,91,95,98,99,100,102,104]. In one case, denosumab was administered due to a misdiagnosis of a tumor-to-tumor metastasis of breast cancer to a giant cell tumor of bone [99]. Interestingly, the patient showed radiological improvement in her disease. Radiotherapy was provided in 34/45 [72,74,79,90,91,95,100,101,102,104,106] (75.6%) patients. In 3/45 (6.6%) [76,77,92] cases patients did not receive adjuvant treatment.

#### 3.2.6. Outcome

Follow-up information was available for 79/153 (51.6%) [65,67,68,69,70,71,72,74,75,77,79,83,88,89,90,92,93,95,98,99,100,104,106] patients. Follow-up time ranged from 3 to 180 months (median 19.5). In 69/79 (87.3%) [65,67,68,69,70,71,72,74,75,77,79,83,88,89,90,92,93,95,98,100,104,106] cases patients were alive with no evidence of recurrence or metastasis, 5/79 (6.3%) [65,68] were alive with disease, 4/79 (5.2%) [65,99] succumbed to disease 1/79 (1.2%) [65] died of unknown cause.

### 3.3. Signet-Ring Cell Carcinoma

#### 3.3.1. Demographic, Clinical and Pathological Features

Signet-ring cell carcinomas (SRCCs) display a signet-ring cell or signet-ring cell-like morphology that can be encountered in a variety of epithelial tumors. This morphology occurs more commonly in breast and stomach carcinomas, followed by colon, pancreas, bladder, prostate, and mesothelioma. Furthermore, several other tumors may occasionally show a signet-ring cell appearance, including mesenchymal tumors (smooth muscle tumors and ovarian stromal tumors), central nervous system tumors (oligodendrogliomas), melanomas, and lymphomas [159,160].

SRCC-B was first described in 1941 by Saphir and was classified as a distinct tumor subtype until 2003 by WHO [106]. It is an extremely rare primary breast carcinoma characterized by the presence of signet-ring cell morphology in a significant proportion of tumor cells. Some previous studies have required an amount of 20% of tumor cells to have signet-ring morphology for SRCC-B diagnosis [106]. Apart from SRCC-B, there is a variety of breast tumors that may display focal or diffuse signet-ring cell morphology, including invasive lobular carcinoma, lobular carcinoma in situ, invasive mucinous carcinoma, and solid papillary carcinoma. SRCC-Bs are considered highly aggressive cancers with a worse outcome than other types of breast carcinoma [106].

In a recent study, SRCC-B represents less than 1% of all breast carcinomas, accounting for 0.04% [130]. In our review, we found 27 articles describing 90 cases of SRCC. The mean age was 58.3 years (range 32–86 years). The mean tumor size was 47.2 mm (range 10–200). Histological subtype was reported in 90/90 (100%) [107,108,109,110,111,112,113,114,115,116,117,118,119,120,121,122,123,124,125,126,127,128,129,130,131,132,133] cases. Features of ductal (invasive breast carcinoma of no special type) differentiation were present in 35/90 (38.9%) [108,109,110,111,112,114,115,116,117,118,119,120,122,124,128,129,133], while 26/90 (28.9%) [107,110,114,121,125,126,127,131,132,133] were invasive lobular carcinomas, 2/90 (2.2%) [123,133] had mucinous differentiation and 27/90 (30%) [110] were pure SRCCs. The demographic, clinicopathological and treatment features of the reported cases are displayed in Supplementary Tables S5 and S6.

#### 3.3.2. Imaging Findings

On mammography, SRCC-Bs may appear as an irregularly shaped [132] or lobulated [125] mass. Occasionally, no mass can be detected [116]. In a recent study, around a third of cases of SRCC-Bs presented malignant calcifications [130].

#### 3.3.3. Histological Findings and Differential Diagnosis

**Figure 7:**
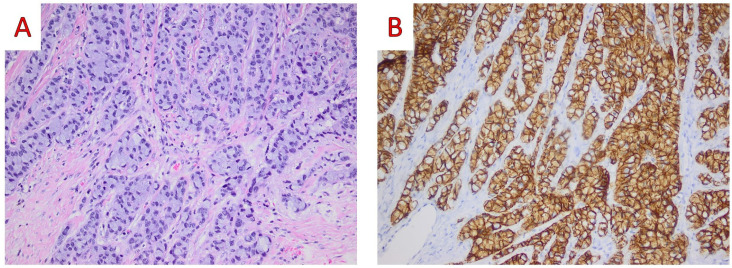
(**A**) On medium power examination, tumor cells show a pure signet ring cell morphology with displacement of the cell nucleus (Hematoxylin and Eosin, H&E; ×200); (**B**) On immunohistochemical examination positive immunostaining for E-cadherin reveals the ductal nature of the carcinoma (E-cadherin mouse monoclonal NCH-38, Dako ×200) (original, previously unpublished photos).

SRCC-Bs display abundant intracellular mucin, displacing the nucleus and giving the characteristic signet-ring cell appearance. The amount of signet-ring cells varies among different publications but the majority requires at least 20% of tumors cell to have this morphology [106]. Pure SRCC-Bs are composed entirely of signet-ring cells. Only 12/90 (13.6%) [122,125,128,133] cases reported histological grade, with 3/12 (25%) [133] being grade I, 5/12 (41.7%) [125,128,133] being grade II, and 4/12 (33.3%) [122,128,133] being grade III. The lymph node status was reported in 72/90 (80%) [108,109,110,111,112,115,116,117,118,119,122,123,124,125,128,130,133] cases. Positive lymph nodes were documented in 46/72 (59.7%) [108,109,110,112,115,116,119,122,124,128,130,133] patients. Figure 7 displays a case of SRCC-B with ductal phenotype.

The differential diagnosis of SRCC-Bs includes metastatic SRCCs from the gastrointestinal tract or other organs. Metastatic gastrointestinal tract carcinomas lack a ductal or lobular carcinoma in situ component, and immunohistochemically, they are positive for CDX-2 and negative for GATA-3, mammaglobin, GCDFP-15, estrogen, and progesterone receptors.

#### 3.3.4. Molecular Studies

To the best of our knowledge there have been no molecular studies for this rare tumor type.

#### 3.3.5. Treatment

In 47/90 (52.2%) [109,111,112,113,115,116,117,118,119,121,122,123,124,125,126,127,128,129,130,131,132] patients surgical treatment details were reported. In 38/47 (81%) [109,111,115,116,117,118,119,121,124,126,129,130] cases, treatment consisted of modified radical mastectomy, radical mastectomy, or simple mastectomy, while 3/47 (6.4%) [122,123,125] patients have treated with breast-conserving surgery. In 2/47 (4.2%) [113,131] cases, a biopsy was performed, and in 4/47 (8.5%) [112,127,128,132], no surgical procedure was performed either due to advanced stage (IV) or due to patient refusal. Details regarding adjuvant treatment were available in 44/90 (48.9%) [109,112,113,115,116,118,121,122,123,124,125,126,127,128,129,130,131,132] cases. Chemotherapy either adjuvant or neoadjuvant was offered in 38/44 (86.3%) [113,115,116,118,122,123,124,125,126,128,129,130,132] cases, hormonal therapy in 5/44 (11.4%) [115,116,121,123,124], and radiotherapy in 20/44 (45.4%) [109,112,115,121,122,123,125,128,130] cases. No adjuvant therapy was administered in 4/44 (9.1%) [127,130,131] patients.

#### 3.3.6. Outcome

Follow-up information was available for 78/90 (86.7%) [106,107,108,110,112,113,114,115,116,117,118,121,122,124,129,130,131,132,133] patients. Follow-up time ranged from 0 to 423 months (median 23). In 38/78 (48.7%) [107,108,110,116,117,118,121,129,130,133] cases patients were alive with no evidence of recurrence or metastasis, 6/78 (7.7%) [107,110,113] were alive with disease, 22/78 (28.2%) [107,110,112,115,122,124,130,132,133] succumbed to disease, 7/78 (9%) [110,114,130,131] died of other cause, and 3/78 (3.8%) [106,114] were lost to contact.

### 3.4. Metaplastic Breast Carcinoma with Melanocytic Differentiation

#### 3.4.1. Demographic, Clinical and Pathological Features

MBC is a heterogeneous group of tumors with distinct morphology and variable prognosis, accounting for 0.2–2% of breast carcinomas [18]. Histologically, MBCs are classified into several different subtypes, including low and high-grade adenosquamous carcinoma [161,162], fibromatosis-like carcinoma [163,164], MBC with squamous cell differentiation (mixed or pure) [165,166,167], sarcomatoid [168,169,170] and heterologous mesenchymal differentiation (matrix-producing) carcinomas [171,172].

A small number of cases of primary tumors with combined histological findings of carcinoma and melanoma have been described in the breast. Most authors describing these tumors consider them a rare variant of MBC. However, the latest edition of the WHO classification of breast tumors does not classify these tumors in the section on metaplastic carcinomas. It considers them a special histologic (melanotic) pattern of IBCs of NST [173].

Similar tumors have been described in the skin [174,175], oral cavity [176], maxillary antrum [177], lung [178], endometrium [179,180], and urinary bladder [181].

In the seven cases we found patient age ranged from 37 to 72 years (mean 49.7). Six cases (85.7%) [134,136,137,138,139] reported tumor size. The mean tumor size was 40 mm (range 20–80). In 6/7 (85.7%) [134,136,137,138,139] patients, nodal status was reported. Three cases (50%) [134,136,139] showed nodal involvement. The demographic, clinicopathological and treatment features of the seven cases are displayed in Supplementary Tables S7 and S8.

#### 3.4.2. Imaging Findings

These tumors appear as a well-circumscribed mass on mammography [134,137] and a solid hypoechoic lesion on ultrasound [137,138].

#### 3.4.3. Histological Findings and Differential Diagnosis

On gross examination, tumors were well circumscribed, either solid or cystic [134], and variegated in color [134,135], tan-white [137], reddish, or brown–black [138]. Connection to the overlying skin was reported only in one case [138].

Microscopically, most tumors showed an admixture of IBC of NST and melanoma features. Multidirectional differentiation was reported by Yen et al. and Noske et al., displaying glandular, squamous, melanocytic, and osseous differentiation in the first [136] and adenoid cystic carcinoma, spindle cell carcinoma and melanocytic differentiation in the second case [137]. Tumors were poorly differentiated with a high mitotic index and, in some cases, with areas of necrosis. Ductal carcinoma in situ (DCIS) was present in four cases [134,135,138].

Electron microscopy showed evidence of epithelial and melanocytic differentiation and a lack of myoepithelial or neuroendocrine features [134,135,136].

Immunohistochemical study showed positive staining with markers of epithelial (CAM5.2, CKAE1/AE3, EMA) and melanocytic (HMB-45, Melan-A) differentiation in the areas showing morphological features of carcinoma and melanoma, respectively. Interestingly, it has been shown S-100 immunopositivity both by the two components [135]. CD117 was positive in the case with adenoid cystic carcinoma component [137]. One case showed focal positive HMB-45 staining in the DCIS [134]. Estrogen and progesterone receptors and HER-2 were negative in all cases.

The differential diagnosis of MBC with melanocytic differentiation includes collision tumors, cancer-to-cancer metastasis, and carcinoma with melanin pigmentation. Histologically, the presence of DCIS, evidence of morphologic transition, and multidirectional differentiation are against the possibility of a collision tumor or cancer-to-cancer metastasis. Furthermore, immunohistochemical staining of the pigmented cells for HMB-45 is a clue of true melanocytic differentiation.

#### 3.4.4. Molecular Studies

To address the clonal relationship between the carcinoma and melanoma components Bunsei et al. [135] performed polymerase chain reaction (PCR)—based microsatellite analysis and found the same patterns of loss of heterozygosity (LOH) in the carcinoma and melanoma components in the primary and metastatic site as well as in situ carcinoma. These findings suggest that both tumor components share the same clonal origin. Besides, this evidence support that breast carcinoma diverged to melanoma early during carcinogenesis in premalignant lesion.

#### 3.4.5. Treatment

In 6/7 (85.7%) cases, surgical treatment was reported. Five out of six (83.3%) [134,135,136,139] patients underwent modified radical mastectomy, while one (16.7%) [138] case was treated with partial mastectomy. Four authors (57.1%) [134,135,138,139] reported details concerning adjuvant treatment. All cases received chemotherapy, and 3/4 (75%) [135,138,139] received radiotherapy.

#### 3.4.6. Outcome

Follow-up was reported in 4/7 (57.1%) [134,135,138] cases ranging from 12 to 72 months (median 14). During this period, two of the patients were alive without evidence of disease [134,138], one was alive with disease [134], and one died of disease [135].

### 3.5. Cumulative Results

#### 3.5.1. Survival

**Figure 8:**
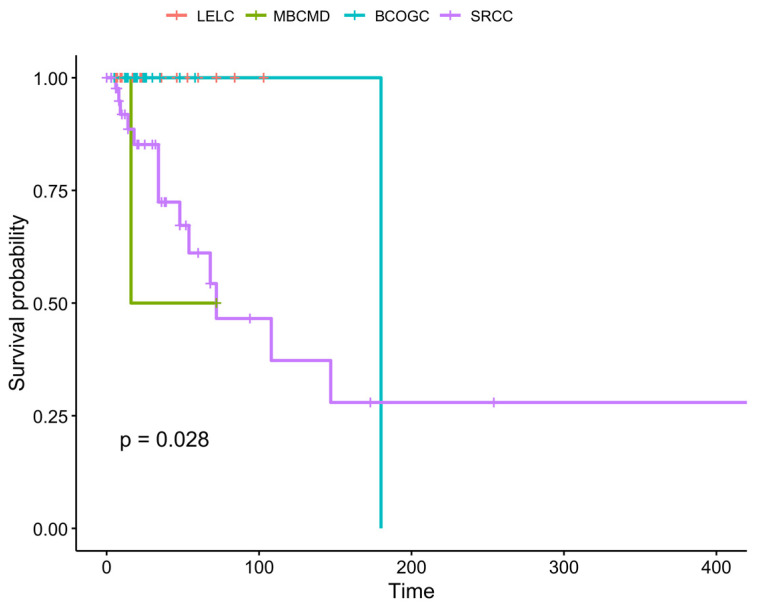
Survival curves for the studied entities. Abbreviations: LELC: Lymphoepithelioma-like breast carcinoma; MBCMD: metaplastic breast carcinoma with melanocytic differentiation; BCOGC: breast carcinoma with osteoclast-like giant cells; SRCC: signet-ring cell carcinoma.

We performed survival analysis for the patients of the four entities (see Figure 8), according to the results, there is statistically significant difference in the survival of the patients (*p* = 0.028). Post hoc analysis showed that (a) patients with LELC had better survival than patients with MBCMD (*p* = 0.0027) (b) patients with LELC had no difference in survival compared to patients with BCOGC (*p* = 1) (c) patients with LELC had better survival from patients with SRCC (*p* = 0.009) (d) patients with MBCMD had worst survival from patients with BCOGC (*p* = 0.0027) but (e) no difference was confirmed when compared to patients with SRCC and finally (f) a marginal significance (*p* = 0.091) was detected when comparing patients with BCOGC with those having SRCC the latter having worst survival probability. Considering survival of patients with LELC and BCOGC, since there was not available monitoring time data we compared the survival percentage, for LELC was 100% and for BCOGC it was 90.7% which leads to statistically confirmed difference (*p* = 0.021).

#### 3.5.2. Clinicopathological Features of the Four Entities

Cumulative results of all entities are presented in Table 1. Differences among the four entities were present for several characteristics, among them patient age, tumor size, treatment approach and as evident from the previous paragraph, survival (see Table 1 for details).

## 4. Conclusions

In summary, we have reviewed the clinical, imaging, pathological, and molecular findings, as well as the treatment and outcome of four extremely rare subtypes of breast carcinoma: LELC-B, BCOGC, SRCC-B, and MBC with melanocytic differentiation. We have also focused on differential diagnosis, which may occasionally be highly challenging. We believe that all the described entities could be recognized as special subtypes in the next edition of the World Health Organization Classification of Breast Tumours.

## Figures and Tables

**Table 1:** Summary table of the main clinicopathological features of the four entities.

	LELC (N = 41)	MBCMD (N = 7)	BCOGC (N = 83)	SRCC (N = 67)	*p*-Value
**Age (years)**					
Mean (SD)	53.0 (8.79)	49.7 (11.7)	50.2 (12.8)	58.3 (11.4)	<0.001
Median [Min, Max]	53.0 [37.0, 69.0]	45.0 [38.0, 72.0]	47.0 [27.0, 84.0]	58.0 [32.0, 86.0]	
**Tumor grade**					
I	0 (0%)	0 (0%)	20 (24.1%)	3 (4.5%)	0.779 †
II	0 (0%)	0 (0%)	18 (21.7%)	5 (7.5%)	
III	0 (0%)	0 (0%)	14 (16.9%)	4 (6.0%)	
Not reported	41 (100%)	7 (100%)	31 (37.3%)	55 (82.1%)	
**Tumor size (mm)**					
Mean (SD)	24.8 (8.42)	40.0 (23.0)	26.9 (14.5)	47.6 (37.8)	<0.001
Median [Min, Max]	22.0 [10.0, 45.0]	30.0 [20.0, 80.0]	25.0 [4.00, 87.0]	37.5 [10.0, 200]	
Not reported	0 (0%)	1 (14.3%)	7 (8.4%)	11 (16.4%)	
**Lymph nodes (positive)**					
Mean (SD)	0.629 (1.50)	0.833 (0.983)	0.984 (2.70)	6.03 (10.5)	<0.001
Median [Min, Max]	0 [0, 8.00]	0.500 [0, 2.00]	0 [0, 14.0]	1.50 [0, 46.0]	
Not reported	6 (14.6%)	1 (14.3%)	20 (24.1%)	33 (49.3%)	
**Lymph nodes (total)**					
Mean (SD)	15.9 (9.12)	12.0 (5.96)	15.4 (10.0)	17.5 (9.33)	0.515
Median [Min, Max]	18.0 [1.00, 33.0]	13.0 [2.00, 17.0]	15.0 [1.00, 42.0]	19.0 [2.00, 46.0]	
Not reported	8 (19.5%)	2 (28.6%)	64 (77.1%)	42 (62.7%)	
**Lymph nodes positivity**					
Yes	10 (24.4%)	4 (57.1%)	23 (27.7%)	30 (44.8%)	
No	25 (61.0%)	3 (42.9%)	50 (60.2%)	15 (22.4%)	<0.001 †
Not reported	6 (14.6%)	0 (0%)	10 (12.0%)	22 (32.8%)	
**Type of lymph node dissection**					
ALND	31 (75.6%)	6 (85.7%)	21 (25.3%)	14 (20.9%)	<0.001 †
SLNB	6 (14.6%)	0 (0%)	13 (15.7%)	2 (3.0%)	
None	0 (0%)	0 (0%)	2 (2.4%)	6 (9.0%)	
Not reported	4 (9.8%)	1 (14.3%)	47 (56.6%)	45 (67.2%)	
**pTNM (tumor component)**					
pT1	0 (0%)	1 (14.3%)	26 (31.3%)	0 (0%)	0.295 †
pT2	0 (0%)	4 (57.1%)	31 (37.3%)	0 (0%)	
pT3	0 (0%)	2 (28.6%)	7 (8.4%)	0 (0%)	
pT4	0 (0%)	0 (0%)	1 (1.2%)	0 (0%)	
Not reported	41 (100%)	0 (0%)	18 (21.7%)	67 (100%)	
**Breast preserving surgery**					
No	16 (39.0%)	5 (71.4%)	24 (28.9%)	15 (22.4%)	0.078 †
Yes	23 (56.1%)	1 (14.3%)	16 (19.3%)	7 (10.4%)	
Not reported	2 (4.9%)	1 (14.3%)	43 (51.8%)	45 (67.2%)	
**Radiotherapy**					
No	16 (39.0%)	1 (14.3%)	7 (8.4%)	13 (19.4%)	0.392 †
Yes	17 (41.5%)	3 (42.9%)	11 (13.3%)	8 (11.9%)	
Not reported	8 (19.5%)	3 (42.9%)	65 (78.3%)	46 (68.7%)	
**Chemotherapy**					
Chemotherapy	18 (43.9%)	0 (0%)	4 (4.8%)	12 (17.9%)	<0.001 †
Hormonal therapy	2 (4.9%)	0 (0%)	6 (7.2%)	1 (1.5%)	
Chemotherapy and hormonal therapy	1 (2.4%)	0 (0%)	3 (3.6%)	4 (6.0%)	
Nothing	12 (29.3%)	0 (0%)	4 (4.8%)	3 (4.5%)	
Not reported	8 (19.5%)	7 (100%)	65 (78.3%)	47 (70.1%)	
Chemotherapy and hormonal and immunotherapy	0 (0%)	0 (0%)	1 (1.2%)	0 (0%)	
**Monitoring time (months)**					
Mean (SD)	31.4 (25.8)	28.0 (29.4)	28.4 (34.5)	47.3 (75.1)	0.972
Median [Min, Max]	24.0 [3.00, 103]	14.0 [12.0, 72.0]	19.5 [3.00, 180]	23.0 [0, 423]	
Not reported	13 (31.7%)	3 (42.9%)	59 (71.1%)	21 (31.3%)	
**Life status**					
ANED	29 (70.7%)	2 (28.6%)	36 (43.4%)	27 (40.3%)	<0.001 †
AWD	0 (0%)	1 (14.3%)	3 (3.6%)	6 (9.0%)	
DOD	0 (0%)	1 (14.3%)	4 (4.8%)	16 (23.9%)	
DUC	0 (0%)	0 (0%)	1 (1.2%)	0 (0%)	
DOC	0 (0%)	0 (0%)	0 (0%)	5 (7.5%)	
Not reported	12 (29.3%)	3 (42.9%)	39 (47.0%)	13 (19.4%)	
**Chemotherapy Type**					
Adjuvant	19 (46.3%)	0 (0%)	9 (10.8%)	14 (20.9%)	0.729 †
Neoadjuvant	1 (2.4%)	0 (0%)	1 (1.2%)	2 (3.0%)	
Neoadjuvant and Adjuvant	0 (0%)	0 (0%)	0 (0%)	1 (1.5%)	
Not reported	21 (51.2%)	7 (100%)	73 (88.0%)	50 (74.6%)	

Abbreviations: ALND: Axillary lymph node dissection; ANED: Alive no evidence of disease; AWD: Alive with disease; DOC: Died of other cause; DOD: Died of disease; DUC: Died of unknown cause; LELC: Lymphoepithelioma-like breast carcinoma; Max: maximum; Min: minimum; mm: millimeters; SD: standard deviation; SLNB: Sentinel lymph node dissection. *p*-values for arithmetic data are based on Kruskal Wallis test, comparisons for the categorical data are based on χ^2^ test or Fisher exact test (marked with †).

## Supplementary Materials

The following supporting information can be downloaded at: https://www.mdpi.com/article/10.3390/ijms25158382/s1.
