# Supplementary material for: The “Forgotten” Subtypes of Breast Carcinoma: A Systematic Review of Selected Histological Variants Not Included or Not Recognized as Distinct Entities in the Current World Health Organization Classification of Breast Tumors
Source: Int J Mol Sci. 2024 Aug 1;25(15):8382. doi: 10.3390/ijms25158382 (PMC11313581; doi:10.3390/ijms25158382)
Supplement: Supplementary file 1 [file ijms-25-08382-s001.zip › Supplementary Table S1.pdf]

Authors	Year	Age	Tumor size (mm)	LN status	Surgery	CHT	HT	RT	Outcome (mo)
Kumar et al.	1994	65	20	0	MS, ALND	NM	NM	NM	ANED 7
Cristina et al.	2000	54	15	0/19	WLE, ALND	No	No	Yes	ANED 6
Dadmanesh et al.	2001	43	19	1/1	QE, SLNB	NM	NM	NM	ANED 60
Dadmanesh et al.	2001	53	20	NM	NM	Yes	No	No	ANED 72
Dadmanesh et al.	2001	49	10	0/19	QE, ALND	Yes	No	No	*
Dadmanesh et al.	2001	52	27	0/20	QE, ALND	No	No	No	ANED 36
Dadmanesh et al.	2001	64	20	0/29	MS, ALND	No	No	No	ANED 60
Dadmanesh et al.	2001	69	23	0/19	MS, ALND	No	No	Yes	ANED 48
Naidoo et al.	2001	50	25	2/24	WLE, ALND	NM	No	NM	ANED 3
Pestereli et al.	2002	56	19	2/27	MS, ALND	Yes	No	No	ANED 12
Ilvan et al.	2004	59	35	0/20	WLE, ALND	No	Yes	Yes	ANED 52
Ilvan et al.	2004	67	11	0/16	QE, ALND	No	No	Yes	ANED 46
Sanati et al.	2004	62	30	NR	WLE	NM	NM	NM	NM
Kurose et al.	2005	47	28	0/33	MS, ALND	Yes	Yes	Yes	AWD 19
Saleh et al.	2005	51	20	1/8	WLE, ALND	NM	NM	NM	NM
Kulka et al.	2008	42	25	0/10	WLE, ALND	NM	NM	NM	NM
O'Sullivan Meija et al.	2009	55	20	0/2	Seg. MS, SLNB	Yes	No	Yes	ANED 22
Jeong et al.	2010	37	22	0/13	MS, ALND	Yes	No	No	ANED 23
Kucukzeybek et al.	2011	58	30	0/24	MRM	Yes	No	No	AWD 18
Trihia et al.	2012	53	15	2/30	PM, ALND	NM	NM	NM	NM
Nio et al.	2012	45	30	0/5	QE, ALND	Yes	No	Yes	ANED 12
Dinniwell et al.	2012	55	40	0/2	EB, SLNB	No (patient refused)	No	Yes	ANED 36
Top et al.	2014	59	30	0/23	MS, ALND	Yes	No	No	ANED 9
Abdou et al.	2014	45	20	0/24	QE, ALND	NM	NM	NM	NM

Authors	Year	Age	Tumor size (mm)	LN status	Surgery	CHT	HT	RT	Outcome (mo)
Suzuki et al.	2014	64	20	3+/23	PM, ALND	Yes	No	Yes	ANED 36
Jansari et al.	2015	39	42	NM	NM	NM	NM	NM	Lost
Nankin et al.	2015	39	27	0/5	WLE, ALND	Yes (neoadj.)	Yes	Yes	ANED 24
Herrera-Goepfert et al.	2016	57	21	0	MS, SLNB	Yes	No	No	ANED
Shet et al.	2016	56	30	1+/17	WLE, ALND	Yes	No	Yes	ANED 105
Shet et al.	2016	39	20	(0/18)	MS, ALND	Yes	No	No	AWD 53
Shet et al.	2016	40	25	ND	WLE	Yes	No	Yes	ANED 84
Shet et al.	2016	40	35	NM	MS, ALND	Yes	No	No	ANED 6
Shet et al.	2016	51	30	NM	MS, ALND	Yes	No	No	ANED 24
Sato et al.	2017	50	12	1+/22	LE, ALND	Yes	No	Yes	ANED 10
Abouelfad et al.	2017	66	22	0/10	WLE, ALND	NM	NM	NM	NM
Koufopoulos et al.	2018	57	22	(8+/13)	LE, ALND	Yes	Yes	Yes	ANED
Aridi et al.	2018	62	35	0/11	MRM	No	No	No	ANED 24
Nieto-Coronel et al.	2019	57	40	0/1	MS, SLNB	Yes	Yes	Yes	ANED 17
Fadila et al.	2019	64	15	1+/19	LE, ALND	Yes	No (patient refused)	Yes	ANED 12
Salehiazar et al.	2022	51	20	0	MS, SLNB	Yes	No	Yes	ANED 10
Nanev et al.	2022	49	45	0/17	MRM	No (patient refused)	Yes	No	ANED 25

Supplementary Table S1: Clinicopathological features of lymphoepithelioma-like breast carcinomas.
Abbreviations: ALND: axillary lymph node dissection; ANED: alive with no evidence of disease; AWD: alive with disease; CHT: chemotherapy; DOD: died of disease; EB: excisional biopsy; mm: millimeters; mo: months; LE: lumpectomy; MRM: modified radical mastectomy; MS: mastectomy; neoadj.: neoadjuvant; NM: not mentioned; PM: partial mastectomy; QE: quadrentectomy; RT: radiotherapy; Seg.: segmental; SLNB: sentinel lymph node biopsy; WLE: wide local excision; y: years; *: contralateral LEC 3 years later;
