# Supplementary material for: The “Forgotten” Subtypes of Breast Carcinoma: A Systematic Review of Selected Histological Variants Not Included or Not Recognized as Distinct Entities in the Current World Health Organization Classification of Breast Tumors
Source: Int J Mol Sci. 2024 Aug 1;25(15):8382. doi: 10.3390/ijms25158382 (PMC11313581; doi:10.3390/ijms25158382)
Supplement: Supplementary file 1 [file ijms-25-08382-s001.zip › Supplementary Table S2.pdf]

	Overall<br>(N=41)
**Age (years)**	
Mean (SD)	53.0 (8.79)
Median [Min, Max]	53.0 [37.0, 69.0]
**Tumor size (mm)**	
Mean (SD)	24.8 (8.42)
Median [Min, Max]	22.0 [10.0, 45.0]
**Lymph nodes (positive)**	
Mean (SD)	0.629 (1.50)
Median [Min, Max]	0 [0, 8.00]
Not reported	6 (14.6%)
**Lymph nodes (total)**	
Mean (SD)	15.9 (9.12)
Median [Min, Max]	18.0 [1.00, 33.0]
Not reported	8 (19.5%)
**Lymph nodes positivity**	
No	25 (61.0%)
Yes	10 (24.4%)
Not reported	6 (14.6%)
**Surgery**	
EB, SLNB	1 (2.4%)
LE, ALND	3 (7.3%)
MRM	1 (2.4%)
MS, ALND	12 (29.3%)
MS, SLNB	3 (7.3%)
PM, ALND	2 (4.9%)
QE	1 (2.4%)
QE, ALND	5 (12.2%)
Seg. MS, SLNB	1 (2.4%)

	Overall (N=41)
WLE	2 (4.9%)
WLE, ALND	8 (19.5%)
Not reported	2 (4.9%)
**Radiotherapy**	
No	16 (39.0%)
Yes	17 (41.5%)
Not reported	8 (19.5%)
**Chemotherapy**	
Chemotherapy	18 (43.9%)
Chemotherapy and Hormonal	1 (2.4%)
Hormonal therapy	2 (4.9%)
Nothing	12 (29.3%)
Not reported	8 (19.5%)
**Monitoring (mo)**	
Mean (SD)	31.4 (25.8)
Median [Min, Max]	24.0 [3.00, 103]
Not reported	13 (31.7%)
**Life status**	
ANED	29 (70.7%)
Not reported	12 (29.3%)
**Entity**	
LELC	41 (100%)
**Chemotherapy Type**	
Adjuvant	19 (46.3%)
Neoadjuvant	1 (2.4%)
Not reported	21 (51.2%)

Supplementary Table S2: Analysis of clinicopathological data of lymphoepithelioma-like breast carcinomas.

Abbreviations: ANED: Alive no evidence of disease; LELC: Lymphoepithelioma-like breast carcinoma; ALND: axillary lymph node dissection; EB: excisional biopsy; LE:lumpectomy; Max: maximum; Min:

minimum; mm: millimeters; MRM: modified radical mastectomy; NM: not mentioned; PM: partial mastectomy; QE: quadrentectomy; ; Seg. MS: segmental mastectomy; SD: standard deviation; SLNB: sentinel lymph node biopsy; WLE: wide local excision.
