# Supplementary material for: The “Forgotten” Subtypes of Breast Carcinoma: A Systematic Review of Selected Histological Variants Not Included or Not Recognized as Distinct Entities in the Current World Health Organization Classification of Breast Tumors
Source: Int J Mol Sci. 2024 Aug 1;25(15):8382. doi: 10.3390/ijms25158382 (PMC11313581; doi:10.3390/ijms25158382)
Supplement: Supplementary file 1 [file ijms-25-08382-s001.zip › Supplementary Table S3.pdf]

Authors	Year	Age	Tumor subtype	Tumor grade	Tumor size (mm)	LN status	pTNM	Surgery	RT	Adj. therapy	Outcome (mo)
Agnantis and Rosen	1979	57	Metaplastic - SCC and spindle cell	NM	15	3+	pT1c, N1	NM	NM	NM	DUC
Agnantis and Rosen	1979	50	IC-NST/ILC	NM	5	Negative	pT1a, N0	NM	NM	NM	AWD
Agnantis and Rosen	1979	43	IC-NST/ILC	NM	36	Negative	pT2, N0	NM	NM	NM	DOD
Agnantis and Rosen	1979	84	Metaplastic - SCC and osteosarcomatous differentiation	NM	40	Negative	pT2, N0	NM	NM	NM	ANED
Agnantis and Rosen	1979	48	IC-NST	NM	50	4+	pT2, N2	NM	NM	NM	DOD
Agnantis and Rosen	1979	53	Metaplastic - SCC and spindle cell	NM	40	Negative	pT2, N0	NM	NM	NM	DOD
Agnantis and Rosen	1979	46	IC-NST	NM	36	1+	pT2, N1	NM	NM	NM	ANED
Agnantis and Rosen	1979	49	IC-NST	NM	20	Negative	pT1c, N0	NM	NM	NM	ANED
Levin et al.	1981	53	IC-NST	NM	30	8+/15	pT2, N2	MRM	NM	NM	NM
Sugano et al.	1983	45	IC-NST	NM	20	0+/NM	pT1c, N0	MRM	NM	NM	ANED
Holland et al.	1984	55	IC-cribriform	NM	25	0+/22	pT2, N0	MRM	NM	NM	ANED 58
Holland et al.	1984	54	IC-cribriform	NM	60	0+/20	pT3, N0	MRM	NM	NM	ANED 48
Holland et al.	1984	38	IC-cribriform	NM	40	14+/14	pT3, N3	MRM	NM	NM	AWD 26
Holland et al.	1984	39	IC-cribriform	NM	20	0+/22	pT1c, N0	MRM	NM	NM	AWD 20
Holland et al.	1984	40	IC-cribriform	NM	25	2+/29	pT2, N1	MRM	NM	NM	ANED 19
Holland et al.	1984	32	IC-cribriform	NM	12	10+/15	pT1c, N3	MRM	NM	NM	ANED 13
Kobayashi et al.	1984	59	IC-NST	NM	37	0+/NM	pT2, N0	MRM	NM	NM	ANED 18
Nielsen and Kiaer	1985	56	IC-NST	I	25	0+/NM	pT2, N0	Mastectomy	NM	NM	ANED 24
Nielsen and Kiaer	1985	71	IC-NST	I	10	0+/NM	pT1c, N0	Mastectomy	NM	NM	ANED 18
Nielsen and Kiaer	1985	79	IC-NST/mucinous	III	40	0+/NM	pT2, N0	Mastectomy	NM	NM	ANED 18
Pettinato et al.	1989	40	ILC	NM	20	0+/NM	pT1c, N0	MRM	NM	NM	ANED 14

Authors	Year	Age	Tumor subtype	Tumor grade	Tumor size (mm)	LN status	pTNM	Surgery	RT	Adj. therapy	Outcome (mo)
Athanasou et al.	1989	45	IC-cribriform	I	20	0+/NM	pT1c, N0	Lumpectomy, LN sampling	Yes	No	ANED 12
Stewart and Mutch	1991	82	IC-NST	III	40	Not excised	pT2, Nx	Tru-cut	No	Tamoxifen	NM
Herrington et al.	1994	80	Metaplastic	NM	30	0+/2	pT2, N0	Partial mastectomy/SLNB	Yes	Tamoxifen	ANED 18
Viacava et al.	1995	45	IC-NST	NM	25	0+/42	pT2, N0	MRM	NM	NM	ANED 30
Takahashi	1998	48	ILC	NM	15	0+/NM	pT1c, N0	MRM	No	No	NM
Saimura et al.	1999	41	IC-NST	II	30	0+/NM	pT2, N0	MRM	No	No	ANED 3
Iacocca et al.	2001	46	ILC	NM	109-65	10+/10	pT3, N3	MRM (L) - SM [R]	NM	NM	NM
Gjerdrum et al.	2001	49	IC-NST	NM	32	micrometastases	pT2, N1(mi)	MRM	Yes	CHT	ANED 24
Kishimoto et al.	2002	46	IC-NST	NM	30	0+/NM	pT2, N0	MRM	No	neoadj. CHT	NM
Cai et al.	2004	41	IC-cribriform	I	25	NM	NM	Core biopsy	NM	NM	NM
Vicandi et al.	2004	48	IC-NST	II	45	Yes	NM	NM	NM	NM	NM
Vicandi et al.	2004	42	IC-NST	II	50	Yes	NM	NM	NM	NM	NM
Vicandi et al.	2004	36	IC-NST	II	30	Yes	NM	NM	NM	NM	NM
Vicandi et al.	2004	40	IC-NST	II	35	No	pT2, N0	NM	NM	NM	NM
Vicandi et al.	2004	38	IC-NST	I	12	Yes	NM	NM	NM	NM	NM
Vicandi et al.	2004	47	IC-NST/ILC	I	18	No	pT1c, N0	NM	NM	NM	NM
Vicandi et al.	2004	44	IC-NST	I	17	No	pT1c, N0	NM	NM	NM	NM
Vicandi et al.	2004	46	IC-NST	I	8	No	pT1b, N0	NM	NM	NM	NM
Vicandi et al.	2004	84	IC-NST	II	45	Yes	NM	NM	NM	NM	NM
Lee et al.	2004	72	Metaplastic	III	53	0+/18	pT3, N0	MRM	NM	NM	ANED 25
Sano	2004	46	IC-NST	NM	25	1+/14	pT2, N1	Lumpectomy, LN sampling	NM	NM	NM

Authors	Year	Age	Tumor subtype	Tumor grade	Tumor size (mm)	LN status	pTNM	Surgery	RT	Adj. therapy	Outcome (mo)
Cai et al.	2005	42	IC-cribriform	I	NM	0+/NM	NM	NM	NM	NM	NM
Cai et al.	2005	42	IC-NST	I	24	0+/NM	pT2, N0	NM	NM	NM	NM
Cai et al.	2005	46	IC-NST-cribriform	II	40	0+/NM	pT2, N0	NM	NM	NM	NM
Cai et al.	2005	46	IC-NST-cribriform	II	11	2+/NM	pT1c, N1	NM	NM	NM	NM
Fadare and Gill	2009	43	IC-neuroendocrine	NM	18	NM	NM	NM	NM	NM	NM
Kurokawa et al.	2009	51	IC-pleomorphic	III	13	0+/1	pT1c, N0	Simple mastectomy/SNLB	NM	NM	NM
Shishido-Hara et al.	2010	44	IC-NST	NM	35	0+/NM	pT2, N0	Partial mastectomy/SLNB	NM	NM	NM
Shishido-Hara et al.	2010	83	Metaplastic- spindle cell	III	22	0+/NM	pT2, N0	Partial mastectomy/SLNB	NM	NM	ANED
Jacquet et al.	2010	48	IC-NST	I	15	Positive	NM	NM	NM	NM	ANED
Jacquet et al.	2010	27	IC-NST	II	27	0+/NM	pT2, N0	NM	NM	NM	ANED
Jacquet et al.	2010	48	IC-NST	III	12	0+/NM	pT1c, N0	NM	NM	NM	ANED
Jacquet et al.	2010	53	IC-NST	I	4	0+/NM	pT1a, N0	NM	NM	NM	ANED
Jacquet et al.	2010	34	IC-NST	II	18	Positive	NM	NM	NM	NM	ANED
Jacquet et al.	2010	36	IC-NST	I	18	0+/NM	pT1c, N0	NM	NM	NM	ANED
Jacquet et al.	2010	37	IC-NST	I	40	NM	NM	NM	NM	NM	ANED
Jacquet et al.	2010	49	IC-NST	III	30	NM	NM	NM	NM	NM	ANED
Stratton et al.	2010	36	IC-NST	II	16	3+/8	pT1c, N1	Lumpectomy, LN sampling	Yes	CHT + Tamoxifen	ANED
Richter et al.	2011	64	IC-NST	NM	12-8-6(m)	NM	NM	BCS/SLNB	Yes	CHT + aromatase inhibitor	NM
Jovicic-Milentijevic	2011	40	Adenoid Cystic Ca	I	20	0+/18	pT1c, N0	Skin sparing mastectomy	No	No	ANED 18

Authors	Year	Age	Tumor subtype	Tumor grade	Tumor size (mm)	LN status	pTNM	Surgery	RT	Adj. therapy	Outcome (mo)
Zhou et al.	2014	mean 43.8 years (age range 29 to 70 years).	IC-NST x 31, mixed IC NST-cribriform x 8, mixed IC NST-mucinous x 3	I x 16, II x 26, III x 1.	mean 17 mm (range 10-40)			MRM was the initial therapy in 21 of the 42 patients. Conservative surgery was performed in 14 patients (2 with SNLB and 12 with ALND). Simple mastectomy with SNLB was performed in three patients.	NM	NM	Follow-up data were available for 35 patients. Follow-up time ranged from 1 month to 11 years (mean 46.4 months). All available patients remained alive. Two presented with lung metastasis, respectively 7 and 11 years after initial surgery.
Cozzolino et al.	2014	72	IC-neuroendocrine	NM	17	NM	NM	NM	NM	NM	NM
Albawardi et al.	2014	45	IC-NST	II	30	NM	NM	lumpectomy/SLNB	Yes	hormonal therapy	ANED 20
Zagelbaum et al.	2016	64	IC-NST(a)	NM	40	NM	NM	NM	NM	NM	NM
Ohashi et al.	2017	47	IC-NST	I	23	Positive	NM	NM	NM	NM	NM
Ohashi et al.	2017	50	IC-NST	I	21	Negative	pT2, N0	NM	NM	NM	NM
Ohashi et al.	2017	42	IC-NST	I	30	Positive	NM	NM	NM	NM	NM
Ohashi et al.	2017	48	IC-NST	I	12	Negative	pT1c, N0	NM	NM	NM	NM
Ohashi et al.	2017	43	IC-NST	I	25	Negative	pT2, N0	NM	NM	NM	NM
Peña-Jaimes et al.	2018	72	ILC pleomorphic	III	28	1+/17	pT2, N1	Simple mastectomy/SLNB + ALND	No	Tamoxifen	ANED 24

Authors	Year	Age	Tumor subtype	Tumor grade	Tumor size (mm)	LN status	pTNM	Surgery	RT	Adj. therapy	Outcome (mo)
Turgeman et al.	2018	46	IC-NST	II	60	3+/19	pT3, N1	MRM	No	CHT + Tamoxifen + Denosumab	DOD 180
Güth et al.	2020	51	ILC pleomorphic	III	24	0+/3	pT2, N0	lumpectomy/SLNB	Yes	Tamoxifen	ANED 12
Irelli et al.	2021	49	IC-NST	II	20	0+/3	pT1c, N0	quadrentectomy/SLNB	Yes	CHT	NM
Cyrta et al.	2022	median 46 (range 33-68)	IC-NST x 17	I x 9, II x 7, III x 1	NM	NM	pT1b x 2, pT1c x 9, pT2 x 6	Lumpectomy x 5 Mastectomy x 2	Yes x 16	CHT x 5, HT x 15	NM
Cyrta et al.	2022	median 45 (range 44–48)	IC-mixed x 4	I x 1, II x 3	NM	NM	pT2 x 4	Lumpectomy x 2 Mastectomy x 2	Yes x 3	CHT x 2, HT x 3	NM
Cyrta et al.	2022	median 62 (range 38–84)	Metaplastic x 6	III x 5, unknown x 1	NM	NM	pT1c x 1, pT2 x 5	Lumpectomy x 3 Mastectomy x 3	Yes x 4	CHT x 5	NM
Sajjadi et al.	2022	69	IC-NST	III	NM	Negative	pT1c, N0	NM	NM	NM	NM
Sajjadi et al.	2022	58	IC-NST	II	NM	Negative	pT2, N0	NM	NM	NM	NM
Sajjadi et al.	2022	65	Metaplastic	III	NM	Negative	pT1c, N0	NM	NM	NM	NM
Sajjadi et al.	2022	35	IC-NST	III	NM	Positive	pT4b, N2a	NM	NM	NM	NM
Sajjadi et al.	2022	53	IC-NST	III	NM	Negative	pT3, No	NM	NM	NM	NM
Sajjadi et al.	2022	61	IC-NST	III	NM	Negative	pT3, N0	NM	NM	NM	NM
Angellotti et al.	2022	38	IC-NST	II	6	Negative	pT1b, N0	quadrantectomy/SLNB	Yes	aromastase inhibitor	ANED 35
Angellotti et al.	2022	41	IC-NST	NM	24	Negative	pT2, N0	quadrantectomy/SLNB	Yes	CHT + hormonal therapy	ANED 5

Authors	Year	Age	Tumor subtype	Tumor grade	Tumor size (mm)	LN status	pTNM	Surgery	RT	Adj. therapy	Outcome (mo)
d'Amati et al.	2022	45	IC-NST	II	8	Negative	pT1b, N0	quadrantectomy/SLNB	NM	NM	NM
Wang	2023	48	IC-NST	II	26	Negative	pT2, N0	Partial mastectomy/SLNB	Yes	CHT	ANED

Supplementary Table S3: Clinicopathological features of breast carcinomas with osteoclast-like giant cells.

Abbreviations: ANED: alive with no evidence of disease; AWD: alive with disease; CHT: chemotherapy; DOD: died of disease; mm: millimeters; mo: months; MRM: modified radical mastectomy; N: no; NM: not mentioned; PM: partial mastectomy; RT: radiotherapy; sn: sentinel node; Y: yes; y: years;
