# Supplementary material for: The “Forgotten” Subtypes of Breast Carcinoma: A Systematic Review of Selected Histological Variants Not Included or Not Recognized as Distinct Entities in the Current World Health Organization Classification of Breast Tumors
Source: Int J Mol Sci. 2024 Aug 1;25(15):8382. doi: 10.3390/ijms25158382 (PMC11313581; doi:10.3390/ijms25158382)
Supplement: Supplementary file 1 [file ijms-25-08382-s001.zip › Supplementary Table S4.pdf]

| | Overall (N=83) |
|---|---|
| **Age (years)** | |
| Mean (SD) | 50.2 (12.8) |
| Median [Min, Max] | 47.0 [27.0, 84.0] |
| **Differentiation** | |
| Adenoid Cystic Ca | 1 (1.2%) |
| IC-cribriform | 9 (10.8%) |
| IC-neuroendocrine | 2 (2.4%) |
| IC-NST | 51 (61.4%) |
| IC-NST-cribriform | 2 (2.4%) |
| IC-NST(a) | 1 (1.2%) |
| IC-NST/ILC | 3 (3.6%) |
| IC-NST/mucinous | 1 (1.2%) |
| IC-pleomorphic | 1 (1.2%) |
| ILC | 3 (3.6%) |
| ILC pleomorphic | 2 (2.4%) |
| Metaplastic | 3 (3.6%) |
| Metaplastic- spindle cell | 1 (1.2%) |
| Metaplastic - SCC and osteosarcomatous differentiation | 1 (1.2%) |
| Metaplastic - SCC and spindle cell | 2 (2.4%) |
| **Tumor grade** | |
| I | 20 (24.1%) |
| II | 18 (21.7%) |
| III | 14 (16.9%) |
| Not reported | 31 (37.3%) |
| **Tumor size (mm)** | |
| Mean (SD) | 26.9 (14.5) |
| Median [Min, Max] | 25.0 [4.00, 87.0] |
| Not reported | 7 (8.4%) |

| | Overall (N=83) |
|---|---|
| **Lymph nodes (positive)** | |
| Mean (SD) | 0.984 (2.70) |
| Median [Min, Max] | 0 [0, 14.0] |
| Not reported | 20 (24.1%) |
| **Lymph nodes (total)** | |
| Mean (SD) | 15.4 (10.0) |
| Median [Min, Max] | 15.0 [1.00, 42.0] |
| Not reported | 64 (77.1%) |
| **Lymph nodes positivity** | |
| No | 50 (60.2%) |
| Yes | 23 (27.7%) |
| Not reported | 10 (12.0%) |
| **pTNM (tumor component)** | |
| pT1 | 26 (31.3%) |
| pT2 | 31 (37.3%) |
| pT3 | 7 (8.4%) |
| pT4 | 1 (1.2%) |
| Not reported | 18 (21.7%) |
| **Surgery** | |
| BCS/SLNB | 1 (1.2%) |
| Core biopsy | 1 (1.2%) |
| Lumpectomy, LN sampling | 3 (3.6%) |
| Lumpectomy/SLNB | 2 (2.4%) |
| Mastectomy | 3 (3.6%) |
| MRM | 17 (20.5%) |
| MRM (L) - SM [R] | 1 (1.2%) |
| Partial mastectomy/SLNB | 4 (4.8%) |
| Quadrentectomy/SLNB | 4 (4.8%) |

| | Overall (N=83) |
|---|---|
| Simple mastectomy/SLNB + ALND | 1 (1.2%) |
| Simple mastectomy/SNLB | 1 (1.2%) |
| Skin sparing mastectomy | 1 (1.2%) |
| Tru-cut | 1 (1.2%) |
| Not reported | 43 (51.8%) |
| **Radiotherapy** | |
| No | 7 (8.4%) |
| Yes | 11 (13.3%) |
| Not reported | 65 (78.3%) |
| **Chemotherapy** | |
| Chemotherapy | 4 (4.8%) |
| Chemotherapy and Hormonal | 3 (3.6%) |
| Chemotherapy and Hormonal and Immunotherapy | 1 (1.2%) |
| Hormonal therapy | 6 (7.2%) |
| Nothing | 4 (4.8%) |
| Not reported | 65 (78.3%) |
| **Monitoring (mo)** | |
| Mean (SD) | 28.4 (34.5) |
| Median [Min, Max] | 19.5 [3.00, 180] |
| Not reported | 59 (71.1%) |
| **Life status** | |
| ANED | 36 (43.4%) |
| AWD | 3 (3.6%) |
| DOD | 4 (4.8%) |
| DUC | 1 (1.2%) |
| Not reported | 39 (47.0%) |
| **Entity** | |
| MCOGC | 83 (100%) |

| | Overall (N=83) |
|---|---|
| **Chemotherapy Type** | |
| Adjuvant | 9 (10.8%) |
| Neoadjuvant | 1 (1.2%) |
| Not reported | 73 (88.0%) |

**Supplementary Table S4:** Analysis of clinicopathological features of breast carcinomas with osteoclast-like giant cells.

**Abbreviations:** ALND: axillary lymph node dissection; ANED: alive no evidence of disease; AWD: alive with disease; BCS: breast conserving surgery; Ca: carcinoma; DOD: died of disease; DUC: died of unknown cause; IC: invasive carcinoma; ILC: invasive lobular carcinoma; L: left; LN: lymph node; MRM: modified radical mastectomy; Max: maximum; MCOGC: metaplastic carcinoma with osteoclast-like giant cells; Min: minimum; mm: millimeters; NM: not mentioned; NST: no special type; R: right; SCC: squamous cell carcinoma; SD: standard deviation; SLNB: sentinel lymph node biopsy; SM: simple mastectomy.
