# Supplementary material for: The “Forgotten” Subtypes of Breast Carcinoma: A Systematic Review of Selected Histological Variants Not Included or Not Recognized as Distinct Entities in the Current World Health Organization Classification of Breast Tumors
Source: Int J Mol Sci. 2024 Aug 1;25(15):8382. doi: 10.3390/ijms25158382 (PMC11313581; doi:10.3390/ijms25158382)
Supplement: Supplementary file 1 [file ijms-25-08382-s001.zip › Supplementary Table S5.pdf]

Authors	Year	Differentiation	Age	Tumor size (mm)	Grade	Lymph nodes	Surgery	Adj. therapy	RT	Outcome (mo)
Harris et al.	1978	Ductal	60	15	NM	NP	NM	NM	NM	ANED 6
Harris et al.	1978	Ductal	66	110	NM	NP	NM	NM	NM	ANED 3
Harris et al.	1978	Ductal	80	22	NM	Positive	NM	NM	NM	ANED 10
Al-Hariri	1980	Ductal	56	20	NM	2+/2	MS, ALND	None	Yes	NM
Hull et al.	1980	Pure SRCC	60	20	NM	2+/6	NM	NM	NM	DOD 54
Hull et al.	1980	Pure SRCC	51	100	NM	23+/23	NM	NM	NM	DOD 14
Hull et al.	1980	Pure SRCC	78	90	NM	3+/3	NM	NM	NM	AWD 12
Hull et al.	1980	Pure SRCC	58	100	NM	27+/28	NM	NM	NM	AWD 30
Hull et al.	1980	Ductal	76	60	NM	24+/24	NM	NM	NM	DOD 72
Hull et al.	1980	Ductal	48	50 - 30	NM	6+/18 - 6+/NM	NM	NM	NM	DOD 9
Hull et al.	1980	Ductal	58	50	NM	11+/19	NM	NM	NM	DOD 34
Hull et al.	1980	Ductal	67	30	NM	0+/21	NM	NM	NM	DOC 94
Hull et al.	1980	Ductal	54	30	NM	0+/11	NM	NM	NM	DOC 254
Hull et al.	1980	Ductal	74	150	NM	10+/16	NM	NM	NM	AWD 12
Hull et al.	1980	Ductal	41	50	NM	5+/10	NM	NM	NM	ANED 36
Hull et al.	1980	Ductal	80	40	NM	3+/16	NM	NM	NM	ANED 173
Hull et al.	1980	Ductal	64	60	NM	1+/19	NM	NM	NM	ANED 32
Hull et al.	1980	Ductal	56	40	NM	0+/19	NM	NM	NM	ANED 60
Hull et al.	1980	Ductal	50	50	NM	2+/10	NM	NM	NM	ANED 6
Hull et al.	1980	Lobular	63	30	NM	0+/20	NM	NM	NM	DOC 0
Hull et al.	1980	Lobular	73	60	NM	21+/21	NM	NM	NM	DOD 18
Hull et al.	1980	Lobular	69	50	NM	2+/12	NM	NM	NM	DOD 147
Hull et al.	1980	Lobular	49	NM	NM	0+/10	NM	NM	NM	DOD 108
Hull et al.	1980	Lobular	66	NM	NM	0+/25	NM	NM	NM	DOD 34
Hull et al.	1980	Lobular	53	40	NM	10+/NM	NM	NM	NM	AWD 6

Authors	Year	Differentiation	Age	Tumor size (mm)	Grade	Lymph nodes	Surgery	Adj. therapy	RT	Outcome (mo)
Hull et al.	1980	Lobular	55	20	NM	0+/25	NM	NM	NM	ANED 21
Hull et al.	1980	Lobular	45	80/20	NM	1+/16-2+/30	NM	NM	NM	ANED 38
Hull et al.	1980	Lobular	66	10	NM	46+/46	NM	NM	NM	ANED 3
Yoshida et al.	1992	Ductal	68	30	NM	Negative	MRM	NM	NM	NM
Ruiz et al.	1997	Ductal	64	NM	NM	Positive	None	NM	Yes	DOD
Kennebeck and Alagoz	1998	Ductal	69	20	NM	1+/12	MRM	CHT + tamoxifen	Yes	DOD 48
Izuishi et al.	1999	Ductal	52	25	NM	2+/26	MRM	CHT + tamoxifen	No	ANED 48
Liu and Chen	2000	Ductal	68	10	NM	Negative	MRM	NM	NM	ANED 6
Colak et al.	2005	Ductal	40	NM	NM	Negative	MRM + pelvic mass debulging + right oophorectomy	Neoadj. CHT + CHT	No	ANED 8
Srinivas et al.	2005	Ductal	70	55	NM	10+/14	MRM	NM	NM	NM
Hara et al.	2010	Ductal	74	13	3	Positive	BCS/SLNB	CHT at recurrence	Yes	DOD
Leung et al.	2011	Mucinous	39	18	NM	Negative	Lumpectomy/SLNB	CHT + tamoxifen	Yes	NM
Ertas et al.	2012	Ductal	54	NM	NM	Positive	MRM	CHT + tamoxifen	No	DOD 68
Karabagli and Kilic	2013	Lobular	48	15	2	Negative	Lumpectomy	CHT	Yes	ANED 20
Chatterjee et al.	2017	Ductal	52	40	3	Positive	MRM	CHT	No	NM
Chatterjee et al.	2017	Ductal	32	35	3	Positive	MRM	CHT	No	NM

Authors	Year	Differentiation	Age	Tumor size (mm)	Grade	Lymph nodes	Surgery	Adj. therapy	RT	Outcome (mo)
Chatterjee et al.	2017	Ductal	58	23	2	Positive	None (stage IV disease)	CHT	Yes	NM
Chatterjee et al.	2017	Ductal	50	56	2	Positive	MRM	Neoadj. CHT	No	NM
Chatterjee et al.	2017	Ductal	65	30	2	Positive	MRM	CHT	No	NM
Wang et al.	2020	NM	38 years to 84 years, with a mean age of 53 ± 12 year	12-100, mean diameter was 32 ± 21.2 mm.	NM	Positive in 14 patients	MRM x 16, RM x 6, Mastectomy x1	CHT x 21 / No treatment x 2	Yes x 12	ANED x 11, DOD x 6, DOC x 2, lost x 2
Zheng	2023	Ductal	86	95	1	Positive	NM	NM	NM	DOD 8
Zheng	2023	Lobular	53	50	3	Negative	NM	NM	NM	ANED 68
Zheng	2023	Ductal	56	26	1	Negative	NM	NM	NM	ANED 52
Zheng	2023	Mucinous	60	40	2	Negative	NM	NM	NM	ANED 39
Zheng	2023	Lobular	54	45	1	Positive	NM	NM	NM	ANED 14

Supplementary Table S5: Clinicopathological features of signet-ring cell carcinomas.

Abbreviations: ANED: alive with no evidence of disease; AWD: alive with disease; CHT: chemotherapy; DOC: Died of other cause; DOD: died of disease; mm: millimeters; mo: months; MRM: modified radical mastectomy; N: no; NM: not mentioned; PM: partial mastectomy; RT: radiotherapy; sn: sentinel node; Y: yes; y: years;
