# Supplementary material for: The “Forgotten” Subtypes of Breast Carcinoma: A Systematic Review of Selected Histological Variants Not Included or Not Recognized as Distinct Entities in the Current World Health Organization Classification of Breast Tumors
Source: Int J Mol Sci. 2024 Aug 1;25(15):8382. doi: 10.3390/ijms25158382 (PMC11313581; doi:10.3390/ijms25158382)
Supplement: Supplementary file 1 [file ijms-25-08382-s001.zip › Supplementary Table S6.pdf]

	Overall (N=67)
**Age (years)**	
Mean (SD)	58.3 (11.4)
Median [Min, Max]	58.0 [32.0, 86.0]
**Differentiation**	
Ductal	35 (52.2%)
Lobular	26 (38.8%)
Mucinous	2 (3.0%)
Pure SRCC	4 (6.0%)
**Tumor grade**	
I	3 (4.5%)
II	5 (7.5%)
III	4 (6.0%)
Not reported	55 (82.1%)
**Tumor size (mm)**	
Mean (SD)	47.6 (37.8)
Median [Min, Max]	37.5 [10.0, 200]
Not reported	11 (16.4%)
**Lymph nodes (positive)**	
Mean (SD)	6.03 (10.5)
Median [Min, Max]	1.50 [0, 46.0]
Not reported	33 (49.3%)
**Lymph nodes (total)**	
Mean (SD)	17.5 (9.33)
Median [Min, Max]	19.0 [2.00, 46.0]
Not reported	42 (62.7%)
**Lymph nodes positivity**	
No	15 (22.4%)
Yes	30 (44.8%)

	Overall (N=67)
Not reported	22 (32.8%)
**Surgery**	
BCS/SLNB	1 (1.5%)
Biopsy	2 (3.0%)
Lumpectomy	1 (1.5%)
Lumpectomy/SLNB	1 (1.5%)
Mastectomy	1 (1.5%)
MRM	12 (17.9%)
MRM + pelvic mass debulging + right oophorectomy	1 (1.5%)
MS, ALND	1 (1.5%)
None	2 (3.0%)
None (patient refused)	1 (1.5%)
None (stage IV disease)	1 (1.5%)
Not reported	43 (64.2%)
**Radiotherapy**	
No	13 (19.4%)
Yes	8 (11.9%)
Not reported	46 (68.7%)
**Chemotherapy**	
Chemotherapy	12 (17.9%)
Chemotherapy and Hormonal	4 (6.0%)
Hormonal therapy	1 (1.5%)
Nothing	3 (4.5%)
Not reported	47 (70.1%)
**Monitoring (mo)**	
Mean (SD)	47.3 (75.1)
Median [Min, Max]	23.0 [0, 423]
Not reported	21 (31.3%)

	Overall (N=67)
**Life status**	
ANED	27 (40.3%)
AWD	6 (9.0%)
DOC	5 (7.5%)
DOD	16 (23.9%)
Not reported	13 (19.4%)
**Entity**	
SRCC	67 (100%)
**Chemotherapy Type**	
Adjuvant	14 (20.9%)
Neoadjuvant	2 (3.0%)
Neoadjuvant and Adjuvant	1 (1.5%)
Not reported	50 (74.6%)

Supplementary Table S6: Analysis of clinicopathological features of signet-ring cell carcinomas.

Abbreviations: ALND: axillary lymph node dissection; ANED: alive no evidence of disease; AWD: alive with disease; BCS: breast conserving surgery; DOC: died of other cause; DOD: died of disease; MS: mastectomy; Max: maximum; Min: minimum; mm: millimeter; mo: months; MRM: modified radical mastectomy; NM: not mentioned; SD: standard deviation; SLNB: sentinel lymph node biopsy; SRCC: signet ring cell carcinoma.
