# Supplementary material for: The “Forgotten” Subtypes of Breast Carcinoma: A Systematic Review of Selected Histological Variants Not Included or Not Recognized as Distinct Entities in the Current World Health Organization Classification of Breast Tumors
Source: Int J Mol Sci. 2024 Aug 1;25(15):8382. doi: 10.3390/ijms25158382 (PMC11313581; doi:10.3390/ijms25158382)
Supplement: Supplementary file 1 [file ijms-25-08382-s001.zip › Supplementary Table S7.pdf]

Authors	Year	Age (y)	Tumor size (mm)	LN	Stage	Surgery	CHT	RT	Outcome (mo)
Padmore et al.	1996	44	30	0/13	pT2N0	MRM	Yes	No	ANED 12
Padmore et al.	1996	41	30	2+/12	pT2N1	MRM	NM	NM	AWD 12
Nobukawa et al.	1999	72	NM	NM	pT2N1	MRM	Yes	Yes	DOD 16
Yen et al.	2000	45	55	2+/17	pT3N1	MRM	NM	NM	NM
Noske et al.	2008	51	25	N	pT2N0	NM	NM	NM	NM
Bendic et al.	2009	38	20	0/16	pT1cN0	PM	Yes	Yes	ANED 72
Nzegwu et al.	2015	57	80	1+/2	pT3N1(sn)	MRM	Yes	Yes	NM

Supplementary Table S7: Clinicopathological features of metaplastic carcinomas with melanocytic differentiation.

Abbreviations: ANED: alive with no evidence of disease; AWD: alive with disease; CHT: chemotherapy; DOD: died of disease; mm: millimeters; mo: months; MRM: modified radical mastectomy; NM: not mentioned; PM: partial mastectomy; RT: radiotherapy; sn: sentinel node; y: years;
