# Supplementary material for: The “Forgotten” Subtypes of Breast Carcinoma: A Systematic Review of Selected Histological Variants Not Included or Not Recognized as Distinct Entities in the Current World Health Organization Classification of Breast Tumors
Source: Int J Mol Sci. 2024 Aug 1;25(15):8382. doi: 10.3390/ijms25158382 (PMC11313581; doi:10.3390/ijms25158382)
Supplement: Supplementary file 1 [file ijms-25-08382-s001.zip › Supplementary Table S8.pdf]

	Overall (N=7)
**Age (years)**	
Mean (SD)	49.7 (11.7)
Median [Min, Max]	45.0 [38.0, 72.0]
**Tumor size (mm)**	
Mean (SD)	40.0 (23.0)
Median [Min, Max]	30.0 [20.0, 80.0]
Not reported	1 (14.3%)
**Lymph nodes (positive)**	
Mean (SD)	0.833 (0.983)
Median [Min, Max]	0.500 [0, 2.00]
Not reported	1 (14.3%)
**Lymph nodes (total)**	
Mean (SD)	12.0 (5.96)
Median [Min, Max]	13.0 [2.00, 17.0]
Not reported	2 (28.6%)
**Lymph nodes positivity**	
No	3 (42.9%)
Yes	4 (57.1%)
**pTNM (tumor component)**	
pT1	1 (14.3%)
pT2	4 (57.1%)
pT3	2 (28.6%)
**Surgery**	
MRM	5 (71.4%)
PM	1 (14.3%)
Not reported	1 (14.3%)
**Radiotherapy**	
No	1 (14.3%)

	Overall (N=7)
Yes	3 (42.9%)
Not reported	3 (42.9%)
**Chemotherapy**	
Not reported	7 (100%)
**Monitoring (mo)**	
Mean (SD)	28.0 (29.4)
Median [Min, Max]	14.0 [12.0, 72.0]
Not reported	3 (42.9%)
**Life status**	
ANED	2 (28.6%)
AWD	1 (14.3%)
DOD	1 (14.3%)
Not reported	3 (42.9%)
**Entity**	
MBCMD	7 (100%)

Supplementary Table S8: Analysis of clinicopathological features of metaplastic carcinomas with melanocytic differentiation.

Abbreviations: ANED: alive with no evidence of disease; AWD: alive with disease; DOD: died of disease; Max: maximum; MBCMD: metaplastic breast carcinoma with melanocytic differentiation; mm: millimeters; Min: minimum; mo: months; MRM: modified radical mastectomy; NM: not mentioned; PM: partial mastectomy; SD: standard deviation.
